# Fabrication of core/shell ZnWO_4_/carbon nanorods and their Li electroactivity

**DOI:** 10.1186/1556-276X-7-9

**Published:** 2012-01-05

**Authors:** Hyun-Woo Shim, Ah-Hyeon Lim, Gwang-Hee Lee, Hang-Chul Jung, Dong-Wan Kim

**Affiliations:** 1 Department of Materials Science and Engineering, Ajou University, Suwon 443-749, South Korea; 2 Plant Engineering Center, Institute of Advanced Engineering, Yongin 449-863, South Korea

## Abstract

Carbon-coated ZnWO_4 _[C-ZW] nanorods with a one-dimensional core/shell structure were synthesised using hydrothermally prepared ZnWO_4 _and malic acid as precursors. The effects of the carbon coating on the ZnWO_4 _nanorods are investigated by thermogravimetry, high-resolution transmission electron microscopy, and Raman spectroscopy. The coating layer was found to be in uniform thickness of approximately 3 nm. Moreover, the *D *and *G *bands of carbon were clearly observed at around 1,350 and 1,600 cm^-1^, respectively, in the Raman spectra of the C-ZW nanorods. Furthermore, lithium electroactivities of the C-ZW nanorods were evaluated using cyclic voltammetry and galvanostatic cycling. In particular, the formed C-ZW nanorods exhibited excellent electrochemical performances, with rate capabilities better than those of bare ZnWO_4 _nanorods at different current rates, as well as a coulombic efficiency exceeding 98%. The specific capacity of the C-ZW nanorods maintained itself at approximately 170 mAh g^-1^, even at a high current rate of 3 C, which is much higher than pure ZnWO_4 _nanorods.

## Introduction

Since Poizot et al. reported that select transition metal-based oxides exhibit high capacities [1], new anode materials based on metal oxides have been extensively studied [2,3] as promising alternatives to carbon-based materials used as anode materials in commercial Li-ion batteries [LIBs]. However, in spite of all the research, some challenges to overcome still remain, such as large volume changes during Li^+ ^insertion and extraction.

Tailoring nanostructures is one popular approach for improving the electrochemical performance of these materials, such as cyclic retention and rate capability [4,5]. Thus far, considerable efforts have been devoted to overcome these problems by using the active/inactive composite concepts, including core-shell nanostructures, in which the inactive phase serves as a buffer and partly alleviates mechanical stress caused by the volume change of the active phase [6,7]. Carbon coating can be also derived from this concept because carbon materials are often of low activity. Numerous previous studies have demonstrated carbon coating as an effective route to improve the electrochemical performance of metal oxide-based anode materials for LIBs. However, most of the previous methods for producing carbon-coated materials were limited, using glucose and sucrose as carbon precursors to obtain the carbon-rich polysaccharide, as well as relatively complicated [8-10].

Recently, Hassan et al. [11] have reported carbon-coated MoO_3 _nanobelts using malic acid as a new carbon source. However, other metal oxide-based materials for application to anodes of LIBs are rarely reported although the method of carbon coating using malic acid has been published. Herein, the authors report on a simple preparation of one-dimensional core/shell ZnWO_4 _nanorods with homogeneous carbon coating and their enhanced electrochemical performance versus that of lithium as a new anode material for LIBs. Furthermore, when used as anode materials in LIBs, the carbon-coated ZnWO_4 _nanorods exhibited significantly improved rate capabilities when compared to pure ZnWO_4 _nanorods. The result demonstrates that a suitable carbon coating is an effective strategy to improve the rate capabilities of the oxide-based anode materials in LIBs. From a survey of the literature, this is the first report on carbon-coated ZnWO_4 _nanorods.

## Experimental details

Carbon-coated ZnWO_4 _nanorods were achieved in two stages: first, ZnWO_4 _nanorods as core parts were prepared using a hydrothermal process with adjusting pH values at 180°C for 12 h; this was followed by general washing and drying steps. Zinc nitrate hexahydrate (Zn(NO_3_)_2_·6H_2_O, 15 mM, 99.0%, Aldrich Chemicals, St. Louis, MO, USA) and an equal amount of sodium tungstate dehydrate (Na_2_WO_4_·2H_2_O, 15 mM, 99.0%, High Purity Chemicals, Tarapur, Maharashtra, India) were used as starting materials. The ZnWO_4 _nanorods thus obtained were then coated with carbon. Malic acid (C_4_H_6_O_5_, 99.0%, Aldrich Chemicals, St. Louis, MO, USA) was used as the carbon source. The malic acid was first dispersed in toluene (C_7_H_8_, 99.5%, Alfa Chemicals, Berkshire, UK), and the ZnWO_4 _nanorods obtained from the hydrothermal technique were added to toluene while stirring at room temperature for 2 h. Subsequently, the slurry was dried at 120°C for 4 h and then 180°C for 6 h under vacuum.

The weight fraction of the coated carbon was determined by thermogravimetric analysis [TGA] (model DTG-60 H, Shimadzu, Kyoto, Japan). The crystalline phase of the prepared samples was carried out using powder X-ray diffraction [XRD] (model D/max-2500 V/PC, Rigaku, Tokyo, Japan), and the distinct properties of the carbon-coated sample were confirmed within the wavelength range of 1,250 to 1,650 cm^-1 ^using laser Raman spectrometry (spectrometer model SPEX-1403, SPEX, Seoul, South Korea). The microstructures of the carbon-coated samples were examined using transmission electron microscopy [TEM] (model JEM-2100F, JEOL, Tokyo, Japan). High-resolution transmission microscopy [HRTEM] was performed for further sample analysis. The electrochemical performance of the samples versus that of lithium was measured by means of a multichannel potentiostatic/galvanostatic system (model WBCS 3000, WonATech, Seoul, South Korea). All samples were galvanostatically cycled as anodes and recorded in a voltage window between 0.01 and 3.0 V.

## Results and discussions

**Figure 1:**
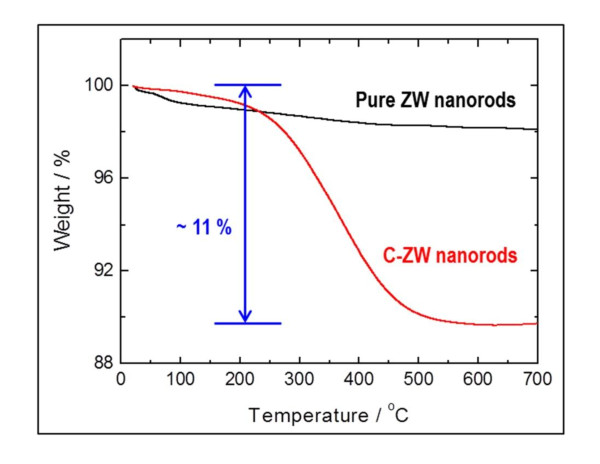
**TGA of pure ZW and C-ZW nanorods**. (By HW Shim et al.).

The obtained powder of 10 wt.% carbon-loaded ZnWO_4 _became dark grey due to the uniform coating. To determine an exact amount of the carbon content in the carbon-coated ZnWO_4 _[C-ZW] nanorods, TGA in air was executed. As shown in Figure 1, the first minor weight-loss step in the temperature range up to 200°C, corresponding to the removal of H_2_O absorbed onto the products, indicated a weight loss of approximately 1% to 2% in all samples prepared in this work. Importantly, the C-ZW nanorods subsequently revealed the highest weight-loss step, but pure ZnWO_4 _[pure ZW] nanorods showed a negligible change in weight loss. These results indicate that combustion of malic acid in the C-ZW nanorods occurred. The combustion reaction begins near 250°C and is completed at approximately 500°C. According to the TGA curves, the carbon content in the product is about 10% after heat treatment of up to 700°C.

**Figure 2:**
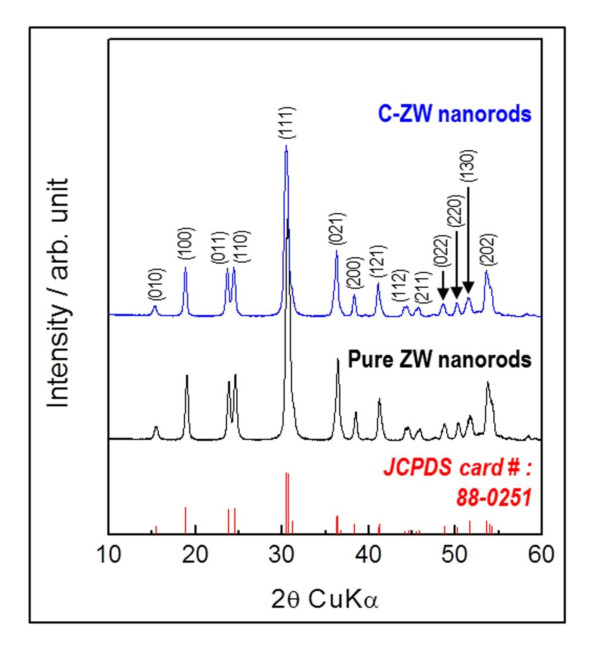
**XRD diffraction patterns of all as-prepared samples**. (By HW Shim et al.).

Figure 2 shows the XRD patterns of the as-prepared pure ZW and obtained C-ZW nanorods. All the reflection peaks of the samples were completely indexed as a highly crystalline, monoclinic, wolframite-tungstate structure, and were in good agreement with the literature values (JCPDS file no.: 88-0251, space group: P2/c) for ZnWO_4 _[12]. No secondary phase other than ZnWO_4 _was detected in any of the products, indicating that the samples obtained were single-phase materials. In particular, as can be seen on the C-ZW nanorod sample, no carbon peak was identified from the XRD pattern. The carbon was hard to detect by XRD analysis, possibly due to its amorphous nature.

**Figure 3:**
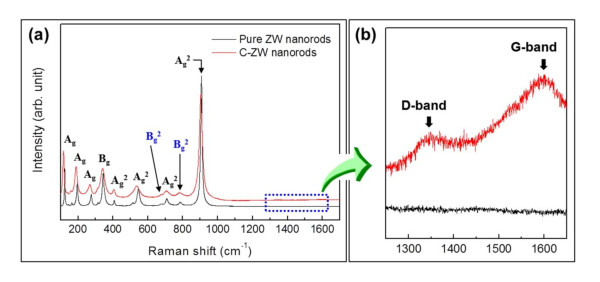
**Raman spectra**. (**a**) Typical Raman spectra of pure ZW and C-ZW nanorods. (**b**) Magnified Raman spectra showing *D *and *G *bands by carbon coating in C-ZW nanorods. (By HW Shim et al.).

In order to clearly confirm a distinct characteristic of carbon properties within the C-ZW nanorods, we investigated the Raman spectra of both the pure ZW and C-ZW nanorods. Figure 3a shows the representative Raman signals (123, 146, 164, 195, 275, 314, 343, 407, 514, 545, 677, 708, 785, and 906 cm^-1^) related to the ZnWO_4 _structure. In particular, the presence of six vibration modes of A_g_^a ^and B_g_^a ^(a = internal stretching modes) should be noted as an important property of monoclinic wolframite ZnWO_4_; the vibration modes arise from the six internal stretching modes caused by each of the six W-O bonds in the WO_6 _octahedrons. In the case of Raman analysis, group theory analysis of wolframite-type ZnWO_4 _predicts 36 lattice modes, of which 18 even vibrations (8A_g _+ 10B_g_) are Raman active [13]. Although all 18 vibration modes were not observed in this Raman spectra, 13 vibration modes were identified exactly, in comparison with previous reports [14,15].

More importantly, the Raman analysis results can confirm the presence of carbon loading in the C-ZW nanorod samples. As depicted in Figure 3b, the spectrum of the C-ZW nanorods in the wavelength range of 1,250 to 1,650 cm^-1^, which is magnified from the dash box in Figure 3a, exhibited obvious differences from the pure ZW nanorods. The peaks indexed by black arrows at approximately 1,370 and 1,580 cm^-1 ^are related to carbon, which are designated in terms of *D *and *G *bands. These peaks are in good correspondence with the Raman spectra of the amorphous carbon reported in the literature [16-18].

**Figure 4:**
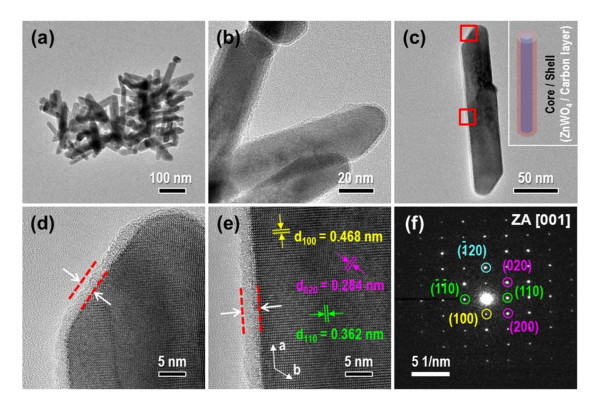
**Representative TEM images of C-ZW nanorods**. (**a**, **b**) Low and high magnifications. (**c**) TEM image of an individual C-ZW nanorod. Inset shows the schematic presentation of a ZnWO_4_/carbon core/shell-structured nanorod. (**d**, **e**) HRTEM images of an individual nanorod in the open-square regions, at the top and side of (c), respectively. (**f**) SAED pattern taken along the <001> zone axis. (By HW Shim et al.).

The TEM observation more clearly demonstrated the success or failure of carbon coating on the C-ZW nanorods. Figures 4a, b show the representative TEM images of the C-ZW nanorods. The obtained morphology of the C-ZW nanorods still maintained the original properties of pure ZW nanorods (core parts) without any visible change. In particular, as expected, from the high-magnification TEM image (Figure 4b), we can observe the carbon layers (shell parts) surrounding the C-ZW nanorods. Moreover, the C-ZW nanorods possess an average diameter of nearly 40 nm and length of 120 to 260 nm, which indicate a relatively large size compared to the pure ZW nanorods.

To further confirm the carbon coating on the surface of ZnWO_4 _nanorods, C-ZW nanorods were studied using TEM, as shown in Figure 4. The C-ZW nanorods obviously sustained the original, rod-like morphology of pure ZW (Figure 4a). A thin carbon layer was homogeneously coated onto the surface of each pure ZW (Figures 4b, c), without deposition of isolated carbon islands by excess carbon pile-up. This resulted in the formation of a hybrid ZnWO_4_/carbon core/shell structure (inset of Figure 4c). The uniform thickness of carbon was approximately 3 nm, based on the HRTEM images (Figures 4d, e) of the individual nanorod, which was taken from the open-square regions in Figure 4c. The surface of the core ZnWO_4 _was very clear and clean, and the magnified view shows the highly crystalline structure of the ZnWO_4 _[19,20]. In Figure 4e, the C-ZW nanorods were structurally uniform, with interplanar spacing of roughly 0.468, 0.362, and 0.284 nm, corresponding to the (100), (110), and (020) lattice spacings of the ZnWO_4 _structure. In addition, the indexed selected area electron diffraction [SAED] pattern via the <001> zone axis revealed the single-crystal nature of the nanorods and further confirmed preferential growth along the [100] direction of the nanorod structures (Figure 4f), as previously reported in the hydrothermal synthesis of pure ZW nanorods [12]. As a result, such uniform carbon loading on ZnWO_4 _is expected to improve the electronic conductivity and electrochemical performance of the pure ZW nanorods.

**Figure 5:**
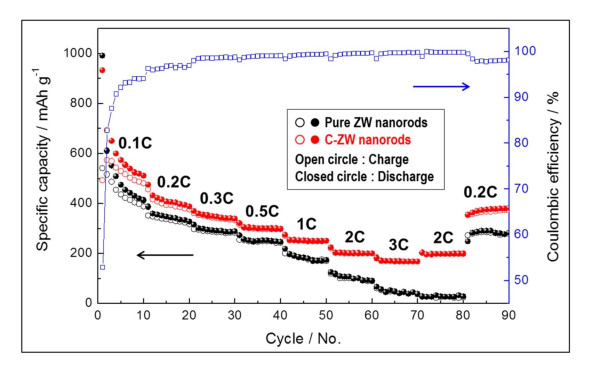
**Rate capabilities**. Comparison of rate capabilities in pure ZW and C-ZW nanorods at different current rates. The open-square curve indicates the coulombic efficiency of the C-ZW nanorods. (By HW Shim et al.).

Figure 5 shows the electrochemical performance as cycling behaviours of the C-ZW and pure ZW nanorod electrodes cycled at different current rates. The cells were first cycled at a current rate of 0.1 C, and after every 10 cycles, the current rate was increased in stages to 3 C. The last 10 cycles proceeded at a current rate of 0.2 C. As predicted, the C-ZW nanorod electrodes exhibited superior rate capabilities compared to pure ZW nanorod electrodes. In particular, at the end of rates 0.1, 0.2, 0.3, 0.5, 1, 2, and 3 C, the C-ZW nanorod electrodes delivered specific capacities of 512, 389, 340, 300, 252, 201, and 169 mAh g^-1^, respectively, while maintaining an excellent coulombic efficiency greater than 98%. Even the specific capacity at a current rate as high as 3 C approached 170 mAh g^-1^, roughly three times higher than that of pure ZW nanorod electrodes. We thus contend that these results can be attributed to the beneficial effects of carbon coating, which enable efficient electronic conductivity and prevent volume expansion during Li-ion insertion and extraction processes.

## Conclusion

In summary, we have demonstrated the synthesis of carbon-coated ZnWO_4 _nanorods with a one-dimensional core/shell structure using a simple hydrothermal route and subsequent carbon coating, and their enhanced Li-storage performance compared with pure ZW nanorods. The uniform loading of amorphous carbon onto the ZnWO_4 _nanorods was clearly confirmed through Raman spectra and HRTEM observations. In particular, the C-ZW nanorods exhibited better capacity delivery than pure ZW nanorods at different current rates and a coulombic efficiency greater than 98%. The specific capacity held steady at approximately 170 mAh g^-1 ^even at a current rate as high as 3 C. Therefore, these C-ZW nanorods may offer an exciting potential for the development of new anode materials for Li-ion batteries.

## Competing interests

The authors declare that they have no competing interests.

## Authors' contributions

H-WS carried out the electrochemical analysis of all as-prepared samples and drafted the manuscript. A-HL carried out the pure ZnWO_4 _and ZnWO_4_/carbon sample preparation. G-HL and H-CJ participated in the microstructural analyses. D-WK designed the study, led the discussion of the results, and participated in writing the manuscript. All authors read and approved the final manuscript.
